# Forty-eight-week efficacy and safety and early CNS tolerability of doravirine (MK-1439), a novel NNRTI, with TDF/FTC in ART-naive HIV-positive patients

**DOI:** 10.7448/IAS.17.4.19532

**Published:** 2014-11-02

**Authors:** Josep M Gatell, Javier O Morales-Ramirez, Debbie P Hagins, Melanie Thompson, Arasteh Keikawus, Christian Hoffmann, Sorin Rugina, Olayemi Osiyemi, Simona Escoriu, Robin Dretler, Charlotte Harvey, Xia Xu, Hedy Teppler

**Affiliations:** 1 Hospital Clinic/IDIBAPS, University of Barcelona, Barcelona, Spain; 2 Clinical Research, Clinical Research Puerto Rico, San Juan, Puerto Rico; 3 Country Health Department, Chatham Country Health, Savannah, USA; 4 AIDS Research Consortium of Atlanta, Atlanta, USA; 5 EPIMED/Vivantes Auguste-Viktoria-Klinikum, Berlin, Germany; 6 ICH Study Center, Hamburg, Germany; 7 Spitalul Clinic de Boli Infectioase, Costanta, Romania; 8 Triple O Research Institute PA, West Palm Beach, USA; 9 Infectious Disease Specialists of Atlanta, Decatur, USA; 10 Merck & Co. Inc, Whitehouse Station, New Jersey, USA

## Abstract

**Introduction:**

Doravirine (DOR) is an investigational NNRTI (aka MK-1439) that retains activity against common NNRTI-resistant mutants. We have previously reported the Part 1 results from a two-part, randomized, double-blind, Phase IIb study in ART-naïve HIV-1-positive patients [1]. At doses of 25, 50, 100 and 200 mg qd, DOR plus open-label tenofovir/emtricitabine (TDF/FTC) demonstrated potent antiretroviral activity comparable to EFV 600 mg qhs plus TDF/FTC and was generally well tolerated at week 24. DOR 100 mg was selected for use in patients continuing in Part 1 and those newly enrolled in Part 2.

**Methods:**

Patients receiving DOR 25, 50 or 200 mg in Part 1 were switched to 100 mg after dose selection. In Part 2, 132 additional patients were randomized 1:1 to DOR 100 mg qd or EFV 600 mg qhs (each with TDF/FTC). We present week 48 efficacy and safety results for all patients in Part 1, and early (week 8) CNS tolerability only for patients randomized to DOR 100 mg or to EFV in Parts 1 and 2 combined. The primary safety endpoint is the % of patients with pre-specified CNS events (all causality) by week 8 for DOR 100 mg qd vs EFV (Parts 1 + 2 combined).

**Results:**

Part 1 week 48 efficacy and safety results are shown below.

The most common DR clinical AEs in the DOR and EFV groups, respectively, were abnormal dreams (10.2%; 9.5%), nausea (7.8%; 2.4%), fatigue (7.2%; 4.8%), diarrhoea (4.8%; 9.5%) and dizziness (3.0%; 23.8%), and were generally mild to moderate.

**Part 1 + 2 Week 8 CNS Event Analysis**: One hundred thirty-two patients were randomized in Part 2, 66 to DOR 100 mg and 66 to EFV. Combining Part 1 and 2, a total of 108 patients received DOR 100 mg and 108 received EFV. By week 8, at least one CNS AE was reported in 22.2% of the DOR group and 43.5% of the EFV group (*p*<0.001). The most common CNS AEs were dizziness (DOR 9.3%; EFV 27.8%), insomnia (6.5%; 2.8%), abnormal dreams (5.6%; 16.7%) and nightmares (5.6%; 8.3%).

**Conclusions:**

In ART-naïve, HIV-1-positive patients also receiving TDF/FTC, DOR 100 mg qd demonstrated potent antiretroviral activity and immunological effect at week 48 and was generally safe and well tolerated. Patients who received DOR 100 mg qd had significantly fewer treatment-emergent CNS AEs by week 8 than those who received EFV.

**Table:** 

Week 48 efficacy (Part 1)			Proportion of patients with virologic response (95% CI)		Mean CD4 change from baseline (95% CI)

Treatment† (mg)		N	HIV RNA <40 c/mL	HIV RNA <200 c/mL	cells/µL
Doravirine qd	25	40	73 (56, 85)	85 (70, 94)	190 (144, 236)
	50	43	72 (56, 85)	74 (59, 87)	134 (78, 190)
	100	42	76 (61, 88)	86 (72, 95)	155 (117, 194)
	200	41	83 (68, 93)	85 (71, 94)	194 (134, 253)
	All	166	76 (69, 82)	83 (76, 88)	168 (143, 193)
Efavirenz qhs	600	42	71 (55, 84)	79 (63, 90)	179 (127, 230)
Missing data approach:				Non-completer=failure	Observed failure

† In combination with TDF/FTC.

**Table:** 

Week 48 safety (Part 1)	Doravirine (*N*=166)	Efavirenz (*N*=42)
	%	%
One or more clinical AEs	88.0	83.3
Drug-related (DR) AEs	36.7	57.1
Serious AE	4.8	9.5
Serious and DR AE	0.0	0.0
Discontinued due to AE	4.2	4.8
Discontinued due to DR AE†	2.4	4.8
Discontinued due to serious AE	0.6	0.0

* All discontinuations due to DR AE occurred by week 24.
